# Qingluoyin granules protect against adjuvant-induced arthritis in rats via downregulating the CXCL12/CXCR4-NF-κB signalling pathway

**DOI:** 10.1080/13880209.2021.1991386

**Published:** 2021-10-25

**Authors:** Min Si, Zheng Ma, Jie Zhang, Xinwei Li, Rui Li, Chao Wang, Huiyu Jia, Shengyong Luo

**Affiliations:** Anhui Academy of Medical Sciences, Hefei, Anhui, China

**Keywords:** Rheumatoid arthritis, adjuvant arthritis, inflammatory cytokine, traditional Chinese medicine

## Abstract

**Context:**

Qingluoyin (QLY) is a traditional Chinese medicine (TCM) formula which has been used in treating human rheumatoid arthritis (RA) for years in China.

**Objective:**

This study investigates the effect of QLY granules on adjuvant arthritis (AA) and the possible mechanism.

**Materials and methods:**

Sprague-Dawley (SD) rats were injected with Complete Freund’s adjuvant (CFA) to induce the AA model. After the onset of arthritis, rats received intragastric administrations of the QLY granules (1.35, 2.70, and 5.40 g/kg) or *Tripterygium* glycosides (TG) tablets (positive drug, 10 mg/kg) for 14 d. After 28 d immunization, the symptoms, inflammatory parameters and molecular mechanisms were investigated.

**Results:**

In the QLY granule (1.35, 2.70, and 5.40 g/kg) therapy groups, the arthritis index decreased to 6.30 ± 2.06, 5.80 ± 1.55, 5.30 ± 1.16 compared with the model (9.00 ± 3.01), paw swelling decreased to 1.56 ± 0.40, 1.28 ± 0.38, 1.12 ± 0.41 mL compared with the model (2.22 ± 0.73 mL). QLY granules (1.35, 2.70 and 5.40 g/kg) significantly reduced the thymus and the spleen indexes, inhibited the production of pro-inflammatory cytokines, and alleviated the pathological changes of joints compared with the model group. Furthermore, the treatment of QLY granules (2.70 and 5.40 g/kg) markedly inhibited CXCL12, CXCR4 (in spleen and synovium) and p-NF-κB p65 (in synovium) protein expression of AA rats.

**Conclusions:**

QLY granules have obvious therapeutic effects on AA rats, which may be associated with downregulating the CXCL12/CXCR4-NF-κB signalling pathway. QLY granules can be used as a candidate for the treatment of RA, which deserves further study.

## Introduction

Rheumatoid arthritis (RA) is a multisystem inflammatory autoimmune disease affecting approximately 1% world population. RA, characterized by intraarticular inflammatory cell infiltration, synovial cell proliferation, cartilage and bone degradation and destruction, can eventually lead to accumulating joint damage, stiffness, deformity, and disability without adequate treatment (Gibofsky 2014). The pathogenesis of RA is complicated, attributable to hyperactive immune and non-immune cells, including fibroblasts, B cells, T cells, macrophages and dendritic cells (DCs) (Chang et al. 2016). These inflammatory cells infiltrate the synovium, promote systemic or local inflammatory progress and increase the production of cytokines [e.g., interleukin (IL)-17, tumour necrosis factor-α (TNF-α), IL-1β, and IL-6], autoantibodies, and matrix metalloproteinase (MMP), resulting in extracellular matrix degradation and bone erosion (Smolen et al. 2018).

Chemokine C-X-C motif chemokine ligand 12 (CXCL12) is released by fibroblast-like synoviocytes (FLS), endothelial cells, periosteum and mesenchymal stem cells (Zhang et al. 2019). C-X-C chemokine receptor type 4 (CXCR4) is a G protein-coupled receptor, which is involved in homing and chemotaxis in the haematopoietic and immune systems as the receptor of CXCL12 (Kawaguchi et al. 2019). Infiltrating immune cells in synoviums, such as T cells and B cells, express the CXCR4 receptor (Buckley et al. 2000). CXCL12 increases in synovium and plasma of RA, involved in synovial hyperplasia, angiogenesis, inflammatory immune cell infiltration, cartilage degradation and bone erosion in RA (Kanbe et al. 2002). Furthermore, CXCL12/CXCR4 axis can directly activate NF-κB pathways (Kim et al. 2015), which plays a critical role in RA. It has been proved that the inflammatory response, synovial cell proliferation and joint damage in patients and laboratory animals of RA could be ameliorated by the inhibition of the NF-κB signalling pathway (Xia et al. 2018; Ibrahim et al. 2020). The CXCL12/CXCR4 axis may thus be a potential therapeutic target for the treatment of RA.

Despite the accessibility to many therapeutic agents at present, such as glucocorticosteroids, nonsteroidal anti-inflammatory drugs (NSAIDs), disease-modifying antirheumatic drugs (DMARDs), and biological drugs (e.g., anti-TNF-therapy) (Liu et al. 2016; Lü et al. 2015), the high cost, as well as undesired side effects, also concern RA patients a great deal, such as immunodeficiency, gastrointestinal disturbances, and humoral disturbances. Therefore, alternative therapies for RA are in urgent need. Traditional Chinese medicine (TCM) has a long history of coping with arthritis, renowned for its low side effect and convenience for long-term rehabilitation treatment in anti-rheumatoid arthritis. Thus, the exploration and development of TCM should be enhanced in terms of its advantages, so as to find new supplementary or alternative medicine for anti-rheumatoid arthritis.

Qingluoyin (QLY) is a traditional Chinese medicine (TCM) formula, which mainly consists of four herbs: *Sophora flavesc*ens Ait. (Leguminosae), Sinomenium *acutum* Rehd. et Wils. (Menispermaceae), *Phellodendron chinensis* Schneid. (Rutaceae), and *Dioscorea spongiosa* J. Q. Xi, M. Mizuno et W. L. Zhao (Dioscoreaceae). Based on the therapeutic theory that emphasizes maintaining and restoring balance in TCM, QLY aims to expel the ‘pathogenetic heat’ in rheumatoid arthritis (RA) and has been successfully applied in treating human RA for years in China (Zuo et al. 2018). The previous studies (Li et al. 2003) found that QLY extract efficiently inhibited angiogenesis and restored the balance of metalloproteinases (MMP)-3 and tissue inhibitor of matrix metalloproteinase (TIMP)-1 in rheumatoid synovium. Experimental evidence (Li et al. 2003) demonstrated that QLY decoction notably alleviated the severity of collagen-induced arthritis (CIA), protecting joints from destruction. At the same time, the clinical trials of QLY (Zhang 2015) in the early period confirmed its ability to ameliorate clinical symptoms. However, the efficacy of QLY granules in RA still calls for verification with increasing research, and the underlying mechanisms need to be further unveiled. In the present study, a pharmacological approach was used to evaluate the anti-arthritic effect of QLY granules and explore the potential mechanisms by employing a rat model of adjuvant-induced arthritis (AA).

## Materials and methods

### Animals

A total of 80 male Sprague–Dawley (SD) rats (150 ± 20 g) were purchased from Nantong University (Nantong, Jiangsu Province, China). Animal care and experimental protocols were approved by the regulations stipulated by the Animal Care Committee of Anhui Academy of Medical Sciences, in accordance with the Guide for the Care and Use of Laboratory Animals.

### Drugs and reagents

QLY granules were prepared by the Institute of Chinese Medicine, Anhui Academy of Medical Sciences. Berberine (batch No. 0713-200107), Oxymatrine (batch No. 110780-200405), Sinomenine (batch No. 0774-200206) were purchased from the National Institute for Control of Pharmaceutical and Biological Products (Beijing, China). *Tripterygium glycosidorum* (TG) tablets (batch No. 2002103B) were purchased from the Zhejiang Deende Medicine Co., Ltd. (Zhejiang, China). Bacillus Calmette-Guerin (BCG) was obtained from REBIO (Shanghai, China). Enzyme-linked immunosorbent assay (ELISA) kits for interleukin (IL)-1β, tumour necrosis factor-alpha (TNF-α), interferon-gamma (IFN-γ), IL-2 and IL-6 were bought from Elisa Biotech (Shanghai, China). Rabbit anti-mouse β-actin, CXCL12, CXCR4, P65, p-P65 were purchased from Affinity Biosciences (Cincinnati, OH, USA).

### Preparations of QLY granules

For the preparation of QLY granules, *Sophora flavescens radix* (350 g), *Sinomenium caulis* (317 g), *Phellodendron chinensis cortex* (317 g), *Dioscorea spongiosae rhizoma* (317 g) were extracted with 10 times the amount of distilled water at boiling temperature for 1 h and then filtered. This procedure was repeated three-times, followed by the ultimate combination of the filtrates. Then the water decoction from the combined extractions was concentrated to a density of 1.2, mixed with 650 g of soluble starch, vacuum dried, and crushed. Afterwards, sucralose (7.5 g) and soluble starch were added to make the total weight reach 1 kg, mixed uniformly, and then granulated.

### Determination of major compounds in QLY granules

The contents of berberine, oxymatrine, sinomenine in QLY granules were determined by high-performance liquid chromatography (HPLC; Thermo U3000, USA) according to the Chinese Pharmacopoeia 2015 edition.

### Induction and treatment of AA

Complete Freund’s adjuvant (CFA) was prepared by suspending heat-killed *Mycobacterium butyricum* in liquid paraffin at 10 mg/mL. AA was induced in SD rats via intradermal injection of 0.1 mL of CFA emulsion into the right hind metatarsal footpad of the rats (Zhang et al. 2019). The rats were randomly divided into six groups: normal, model, QLY (1.35, 2.70, 5.40 g/kg) and TG (10 mg/kg) group (n = 10 per group). After the onset of arthritis on d 15, the rats were given QLY (1.35, 2.70 and 5.40 g/kg) and TG (10 mg/kg) (once per day) for 14 d (from day 15 to day 28) by intragastric administration. An equal volume of vehicle was administered to the normal and model groups simultaneously.

### Evaluation of arthritis

To evaluate the severity of arthritis, arthritis global assessment, arthritis index, and swollen joint count of rats were assessed every three days by two observers blinded to the treatment (Song et al. 2013). The secondary inflammatory paw (left hind) swelling of rats was measured using a water plethysmometer on d 0, 15, 18, 21, 24, and 27. Paw volume (△mL) = paw volume (d 15, 18, 21, 24, 27) – paw volume (d 0) (Chen et al. 2020).

### Thymus index and spleen index

Rats were weighed before being sacrificed on d 28. Then, their thymus and spleen were gently removed and weighed. The thymus or spleen index was calculated as follows: Thymus (or spleen) index = Weight thymus (or spleen) (mg)/Body Weight (g) (Wang et al. 2011).

### Histological examination of spleens and joints

Rats were anesthetized and sacrificed on d 28 after immunization. Secondary knee joints and spleens were removed, fixed in formalin and embedded in paraffin for histopathological analysis. In addition, joints were decalcified in 10% ethylenediaminetetraacetic acid (EDTA) for about one month. The sections were stained with haematoxylin and eosin (H&E) and examined under a fluorescence microscope, and changes in spleens and joints were evaluated histopathologically and analyzed by two blinded observers. To assess the extent of spleen remodelling, five compartments were evaluated, including the cell density lymphatic sheaths, lymphoid follicles, marginal zone, red pulp and the total number of germinal centres (GCs) (Chang et al. 2016). The severity of arthritis was assessed based on pathological changes such as pannus, synovium hyperplasia, cartilage erosion, and infiltrated inflammatory cells. The grading scheme was made up of ordinal categories ranging from 0 (no effect) to 4 (severe effect) (Sultana et al. 2017).

### Cytokine measurement

On d 28 rats were sacrificed and the serum collected from peripheral blood and stored at −80 °C until use. Concentrations of IL-6, IL-1β, IL-2, IFN-γ and TNF-α in serum were measured using ELISA according to the manufacturer’s instructions. The concentration was calculated according to the absorbance (A) at 450 nm.

### Immunostaining analysis

The expressions of CXCL12/CXCR4 and NF-κB were detected by immunohistochemical staining. Sections were incubated with anti-CXCL12, anti-CXCR4, anti-P65 and anti-p-P65 Ab (1:100 dilution) in a humid chamber at 4 °C overnight. After washing with PBS, the sections were incubated with peroxidase anti-goat secondary reagent for 30 min. Subsequently, immunostaining was observed using 3,3-diaminobenzidine (DAB) for 7 min and counterstaining was performed using 0.2% haematoxylin for 45 s at 25 °C and examined under a fluorescence microscope (Axio Scope A1, Germany).

### Protein sample preparation and Western blot analyses

Synovium tissues were homogenized and lsyed with RIPA lysis buffer. Samples were centrifuged at 12,000 rpm for 20 min at 4 °C, followed by the collection of supernatants. Equal amounts of protein samples were mixed with 5× sample buffer (4:1) and heated in boiling water for 10 min. The protein samples were lectrophoresed by sodium dodecyl sulfate-polyacrylamide gel electrophoresis (SDS-PAGE) gel and then electrotransferred onto a polyvinylidene fluoride (PVDF) membrane. The membrane was blocked with 5% non-fat milk for 2 h at 37 °C. After washing with TPBS buffer containing 0.05% Tween-20 for three times, the membranes were then incubated with the primary antibodies (1:1000) of CXCL12 or CXCR4 or P65 or p-P65 overnight at 4 °C. The membranes were washed three times and incubated with horseradish peroxidase-conjugated secondary antibodies (1:10,000) for 2 h at room temperature. Finally, the protein bands were visualized using the enhanced chemiluminescence kit.

### Statistical analysis

All data are expressed as the mean ± S.D. One-way ANOVA with Tukey’s multiple comparison test was applied to determine the differences between groups. The results were considered statistically significant for *p* < 0.05.

## Results

### Quantitative analysis of major compounds in QLY granules

**Figure 1.**
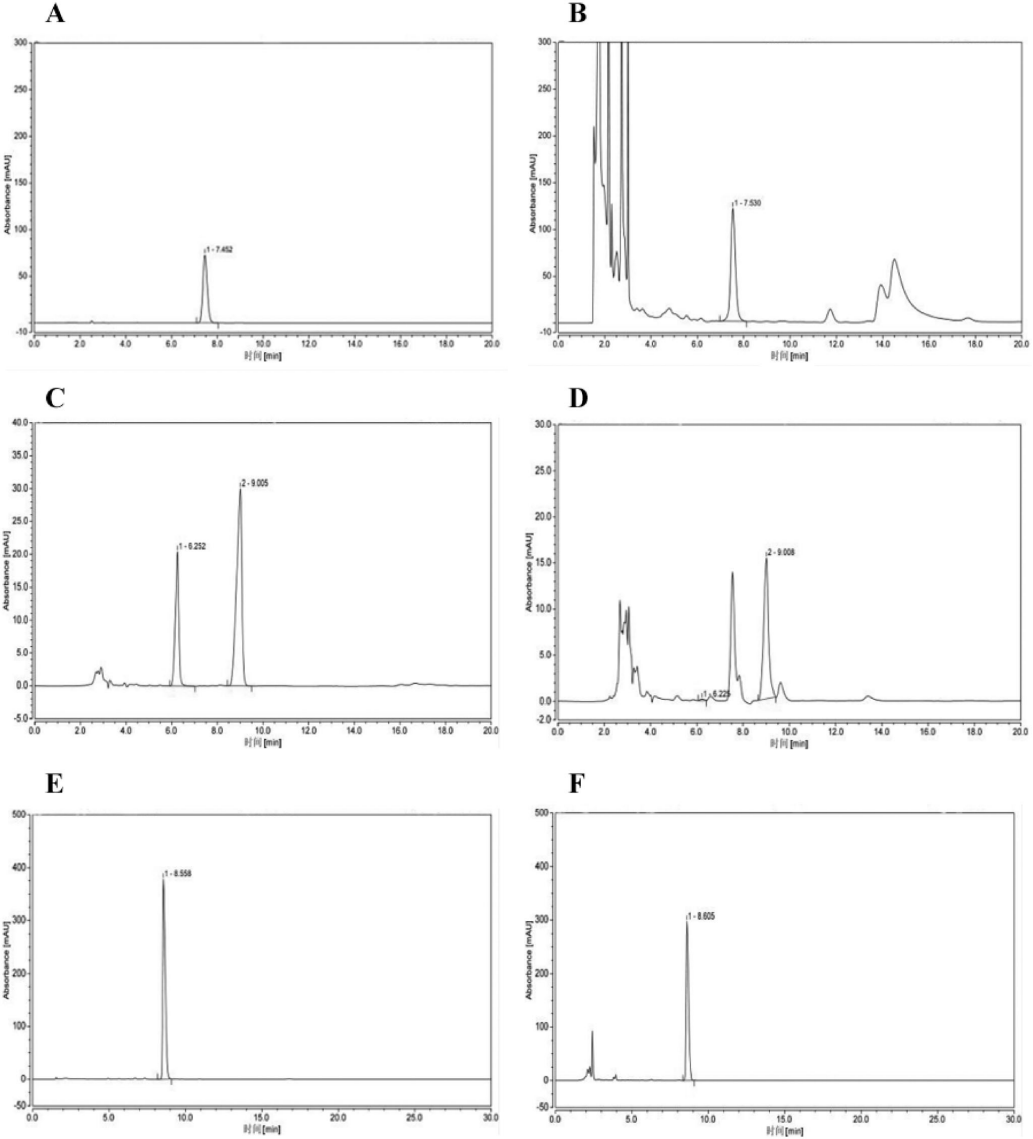
Quantitative analysis of major compounds in QLY granules. HPLC chromatograms. (A) Standard solution of berberine. (B) Extract of Qingluoyin granules. (C) Standard solution of oxymatrine. (D) Extract of Qingluoyin granules. (E) Standard solution of sinomenine. (F) Extract of Qingluoyin granules.

According to the Chinese Pharmacopoeia, berberine, oxymatrine and sinomenine can be used as standards to investigate the quality of *Phellodendron chinensis, Sophora flavescens and Sinomenium acutum,* respectively. Therefore, these three compounds were used as references to verify the composition of QLY. A typical HPLC chromatogram as shown in Figure 1. External standard one-point method was used to calculate the concentrations of berberine, sinomenine, oxymatrine, the results were shown in Table 1.

**Table 1.** Determination results of three ingredients in QLY granules/mg g^−1^.

Ingredient	Concentration (mg/g)
Berberine	10.1
Oxymatrine	4.9
Sinomenine	3.38

### Effects of QLY granules on arthritis global assessment, arthritis index, swollen joint count and paw swelling

**Figure 2.**
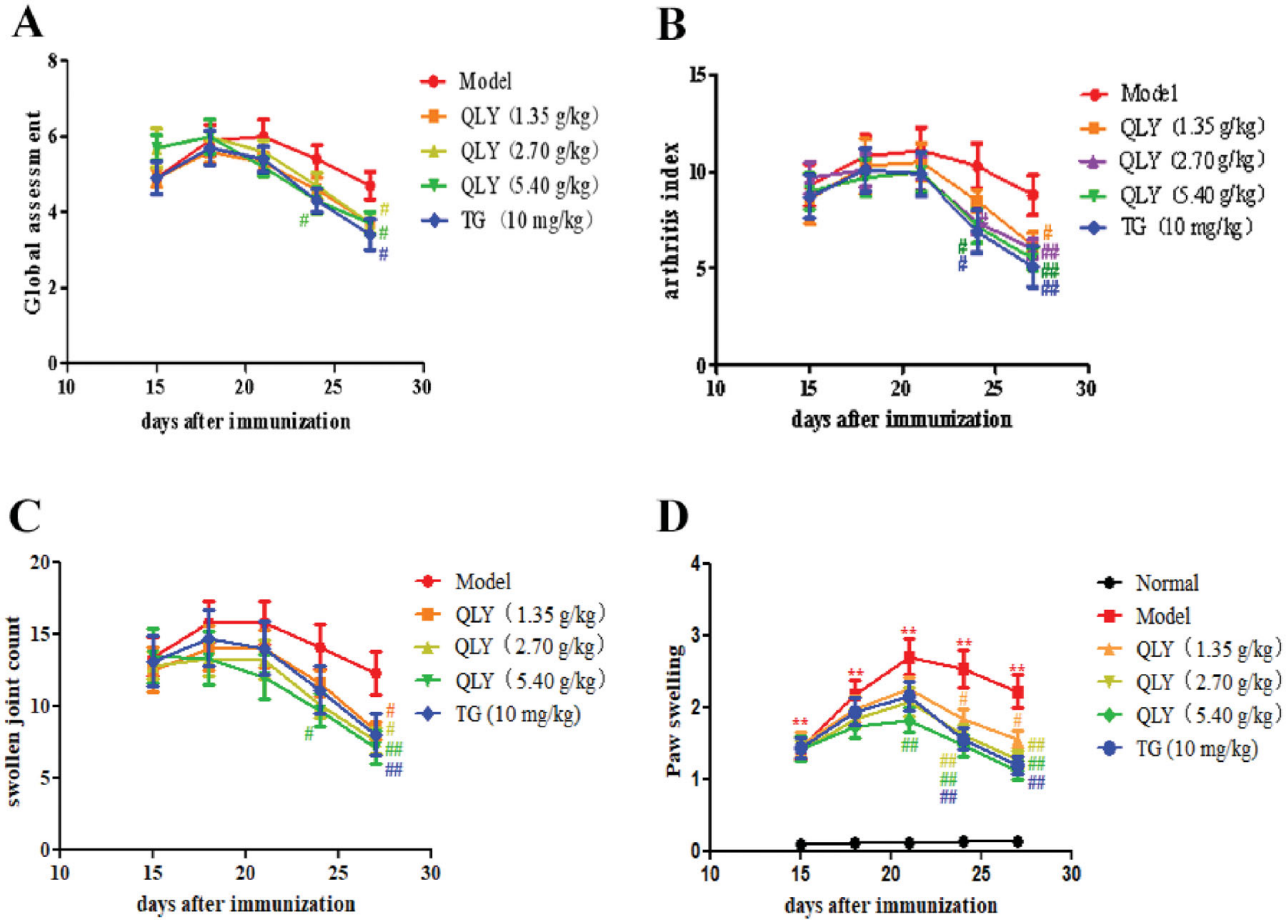
Effects of QLY granules on arthritis signs in AA rats. (A) The global assessment of AA rats. (B) The arthritis index of AA rats. (C) The swollen joint count of AA rats. (D) The paw swelling of AA rats. Data are expressed as the mean ± SD, with 10 animals in each group. **p < 0.01 vs. normal; ^#^*p* < 0.05, ^##^*p* < 0.01 vs. model.

The effects of QLY granules were evaluated using AA, a well-established *in vivo* model of inflammatory joint diseases. The results revealed that QLY granules reduced the global assessment, arthritis index, swollen joint count, and paw swelling. In QLY granules (1.35, 2.70 and 5.40 g/kg) therapy group, the arthritis index decreased to 6.30 ± 2.06, 5.80 ± 1.55 and 5.30 ± 1.16 compared with the model group (9.00 ± 3.01) on day 27, and paw swelling decreased to 1.56 ± 0.40, 1.28 ± 0.38 and 1.12 ± 0.41 mL compared with the model group (2.22 ± 0.73 mL) on day 27 (Figure 2).

### Effects of QLY granules on spleen and thymus index

**Figure 3.**
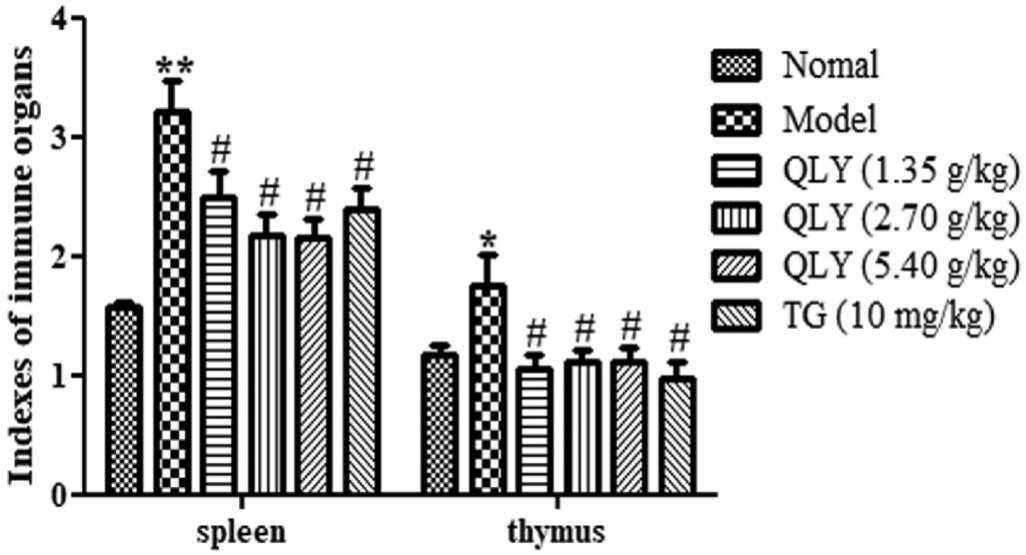
Effects of QLY granules on thymus and spleen indexes. Data are expressed as the mean ± SD, with 10 animals in each group. *p < 0.05, **p < 0.01 vs. normal; ^#^*p* < 0.05 vs. model.

As shown in Figure 3, compared with the normal group (spleen: 1.61 ± 0.63; thymus: 1.18 ± 0.21), the spleen and thymus index of the model group (spleen: 3.21 ± 0.81; thymus: 1.80 ± 0.81) significantly increased. Compared with the model group, QLY granule (1.35, 2.70 and 5.40 g/kg) treatment could significantly reduce the spleen and the thymus index (*p* < 0.05) of AA rats.

### Effects of QLY granules on histopathology of AA joints and spleens

**Figure 4.**
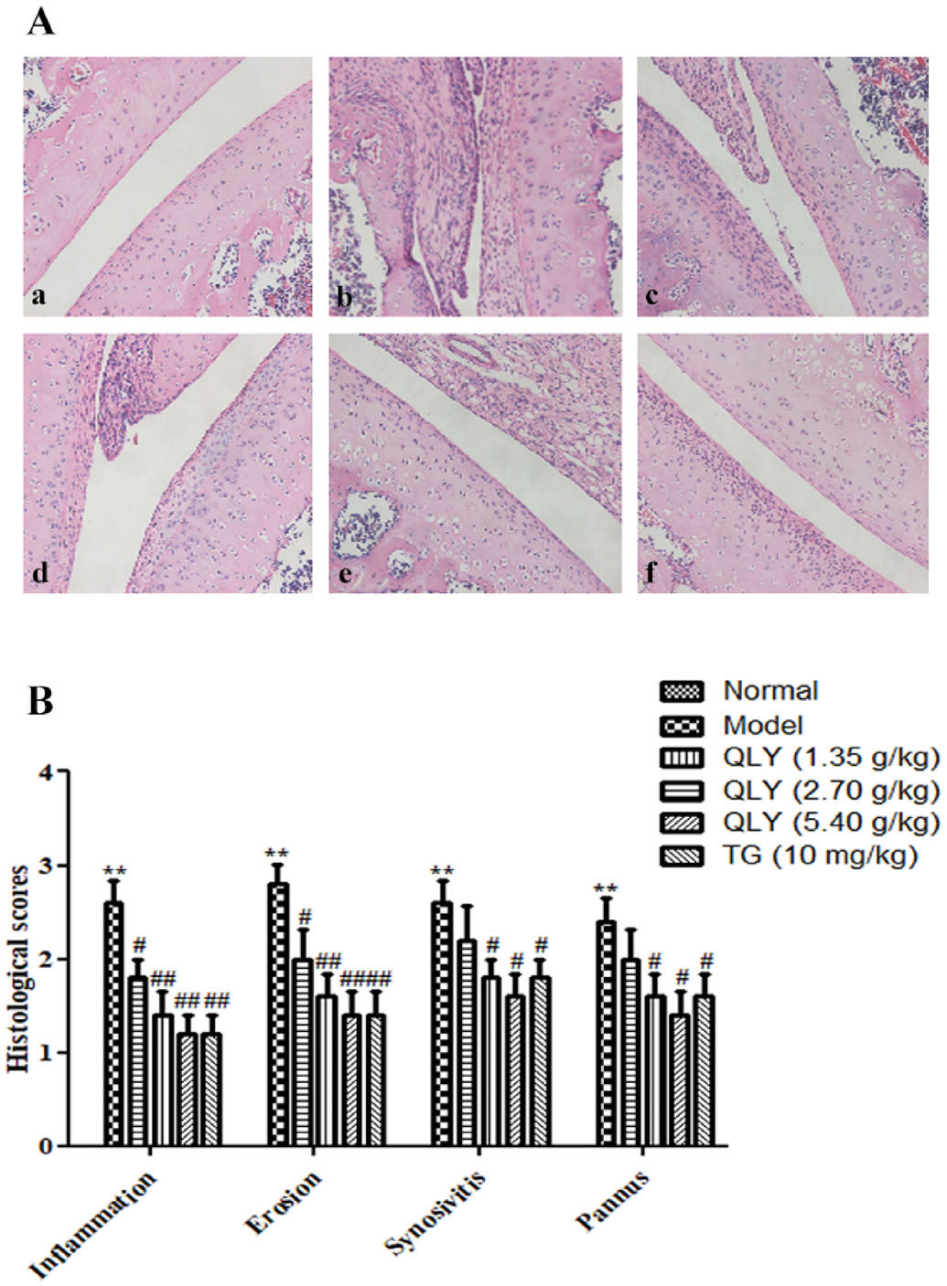
Effects of QLY granules on histopathology of AA joints. The histopathology examinations in joints were observed by H&E staining. (A) Representative histological changes of haematoxylin and eosin-stained sections of the joints (magnification × 400). a: normal; b: model; c: QLY granules (1.35 g/kg); d: QLY granules (2.70 g/kg); e: QLY granules (5.40 g/kg); f: TG (10 mg/kg). (B) Histopathological evaluation of the synovium from the AA rats. The histological appearance was scored for the presence of synovial proliferation, infiltrated inflammatory cells, pannus formation, and cartilage erosion. Data are expressed as the mean ± SD, with 5 animals in each group. **p < 0.01 vs. normal; ^#^*p* < 0.05, ^##^*p* < 0.01 vs. model.

As shown in Figure 4, AA rats developed severe arthritis, which was characterized by marked inflammatory cell infiltration, erosion of articular cartilage and bone, synovial proliferation, and vascular pannus. In comparison, QLY granules (1.35, 2.70 and 5.40 g/kg) could obviously inhibit cellular infiltration and cartilage erosion (*p* < 0.05 or *p* < 0.01).

**Figure 5.**
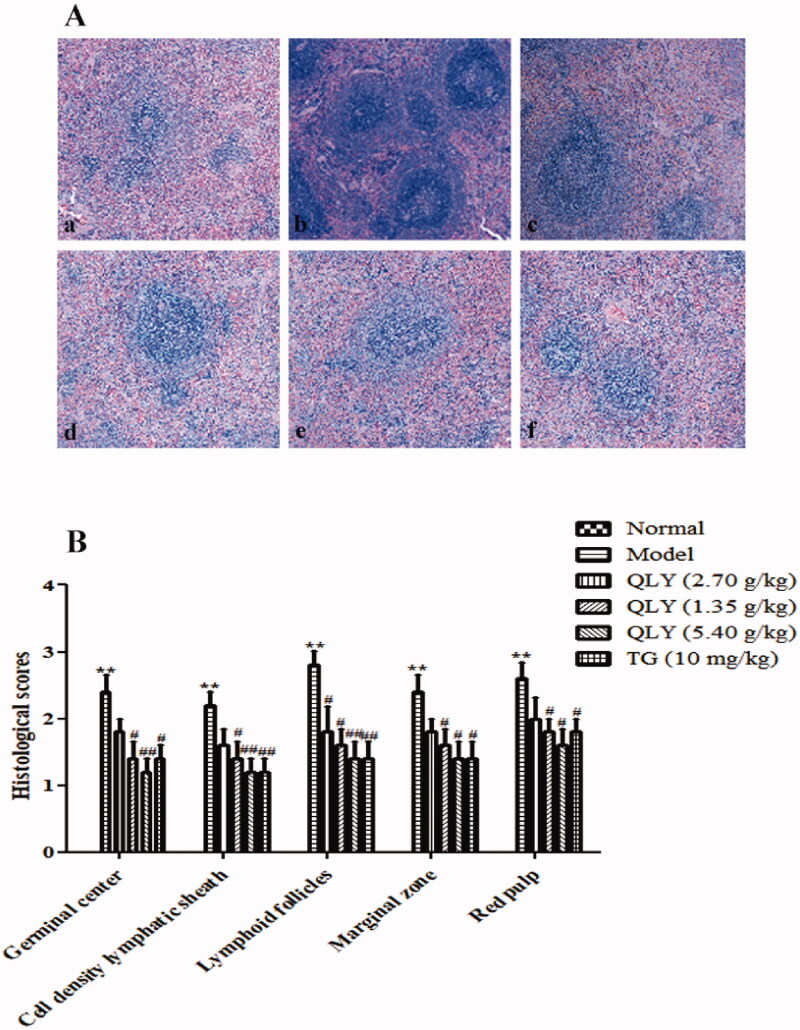
Effects of QLY granules on AA spleen histopathology. (A) Representative micrographs of H&E-stained histological sections of the spleens are shown (magnification × 400). a: normal; b: model; c: QLY granules (1.35 g/kg); d: QLY granules (2.70 g/kg); e: QLY granules (5.40 g/kg); f: TG (10 mg/kg). (B) The histology section shows the cell density lymphatic sheaths, lymphoid follicles, marginal zone, red pulp and the total number of germinal centres (GC). Data are expressed as the mean ± SD, with 5 animals in each group. **p < 0.01 vs. normal; ^#^*p* < 0.05, ^##^*p* < 0.01 vs. model.

Compared with spleen architecture seen in unimmunized animals, increased the total number of germinal centres, cell density lymphatic sheaths, lymphoid follicles, marginal zone, and red pulp was also detected in the model group (*p* < 0.01). However, treatment with QLY granules (2.70, 5.40 g/kg) could significantly decrease the number of germinal centres and the cell density lymphatic sheaths, and alleviate the red pulp congestion (*p* < 0.05 or *p* < 0.01) (Figure 5(A,B)).

### Effects of QLY granules on inflammatory cytokine production in serum

**Figure 6.**
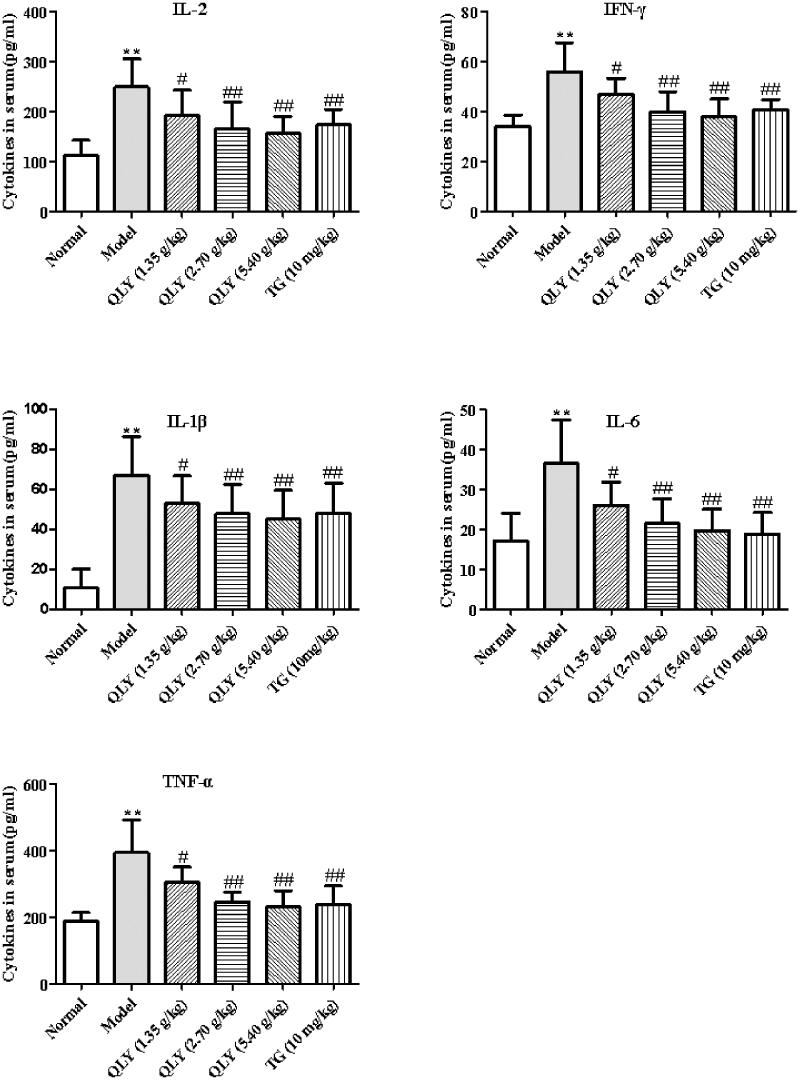
Effects of QLY granules on inflammatory cytokine production in serum. Data are expressed as the mean ± SD, with 10 animals in each group. **p < 0.01 vs. normal; ^#^*p* < 0.05, ^##^*p* < 0.01 vs. model.

The levels of cytokines in serum were detected by ELISA. As shown in Figure 6, exposure to CFA significantly increased the production of serum IL-2, IFN-γ, IL-1β, IL-6 and TNF-α compared with the normal group (*p* < 0.01). However, QLY granules (1.35, 2.70 and 5.40 g/kg) and TG could significantly decrease the levels of IL-2, IFN-γ, IL-1β, IL-6 and TNF-α in serum compared with the model group (*p* < 0.05 or *p* < 0.01).

### Effects of QLY granules on expression of CXCL12/CXCR4 in spleens of AA rats

**Figure 7.**
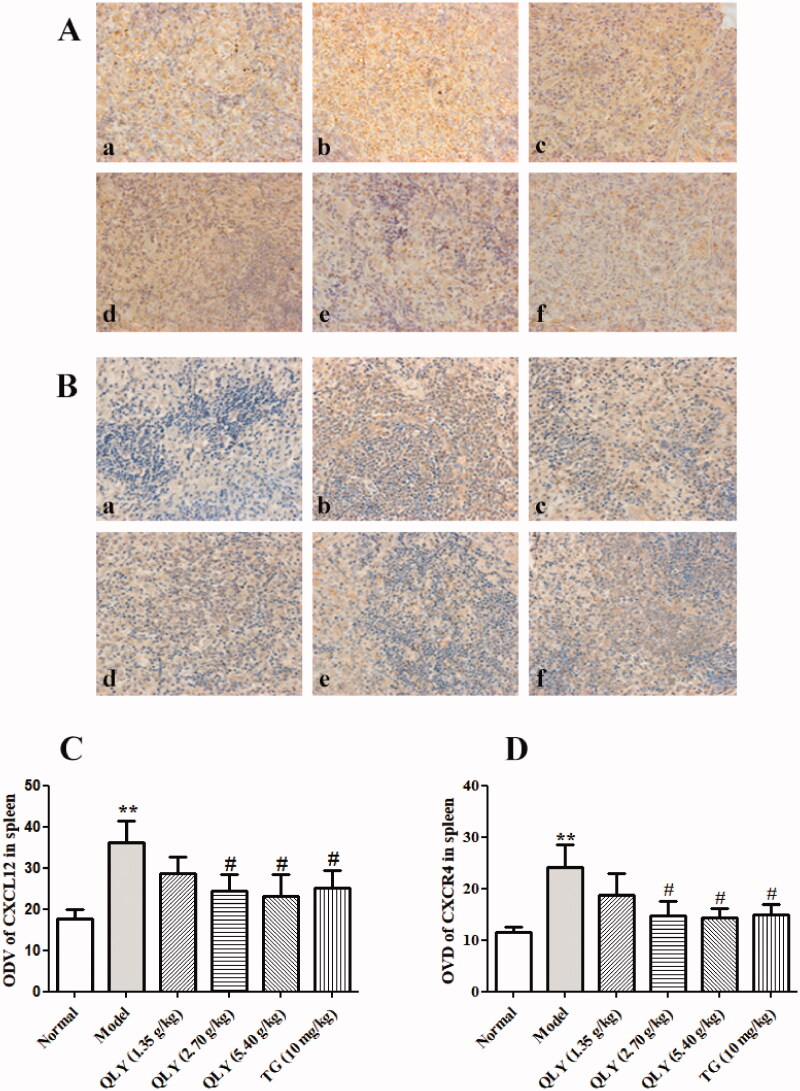
Effects of QLY granules on the expression of CXCL12/CXCR4 in spleen of AA rats. Expression of CXCL12 and CXCR4 level in spleen of AA rats. Representative immunohistochemical analyses of (A) CXCL12 and (B) CXCR4 expression in spleens, illustrating alterations in spleens of each group of rats (magnification, ×400). a: normal; b: model; c: QLY granules (1.35 g/kg); d: QLY granules (2.70 g/kg); e: QLY granules (5.40 g/kg); f: TG (10 mg/kg). Optical density value (ODV) of (C) CXCL12 and (D) CXCR4 in spleens markedly decreased in rats with AA following the administration of QLY granules and TG. Data are expressed as the mean ± SD, with 5 animals in each group. **p < 0.01 vs. normal, ^#^*p* < 0.05 vs. model.

The expression of CXCL12 and CXCR4 proteins in spleens were detected by immunohistochemical staining. As shown in Figure 7, the results demonstrated that CXCL12/CXCR4 expression in model group (ODV: 36.18 ± 5.23, 24.21 ± 4.42, respectively) increased compared with that in the normal group (ODV: 17.64 ± 2.35, 11.44 ± 1.12, respectively). QLY granules (2.70, 5.40 g/kg) could remarkably decrease the positive expression of CXCL12/CXCR4 in spleens of AA rats (*p* < 0.05).

### Effects of QLY granules on expression of CXCL12-mediated inflammatory signalling pathways in synovial tissues of AA rats

**Figure 8.**
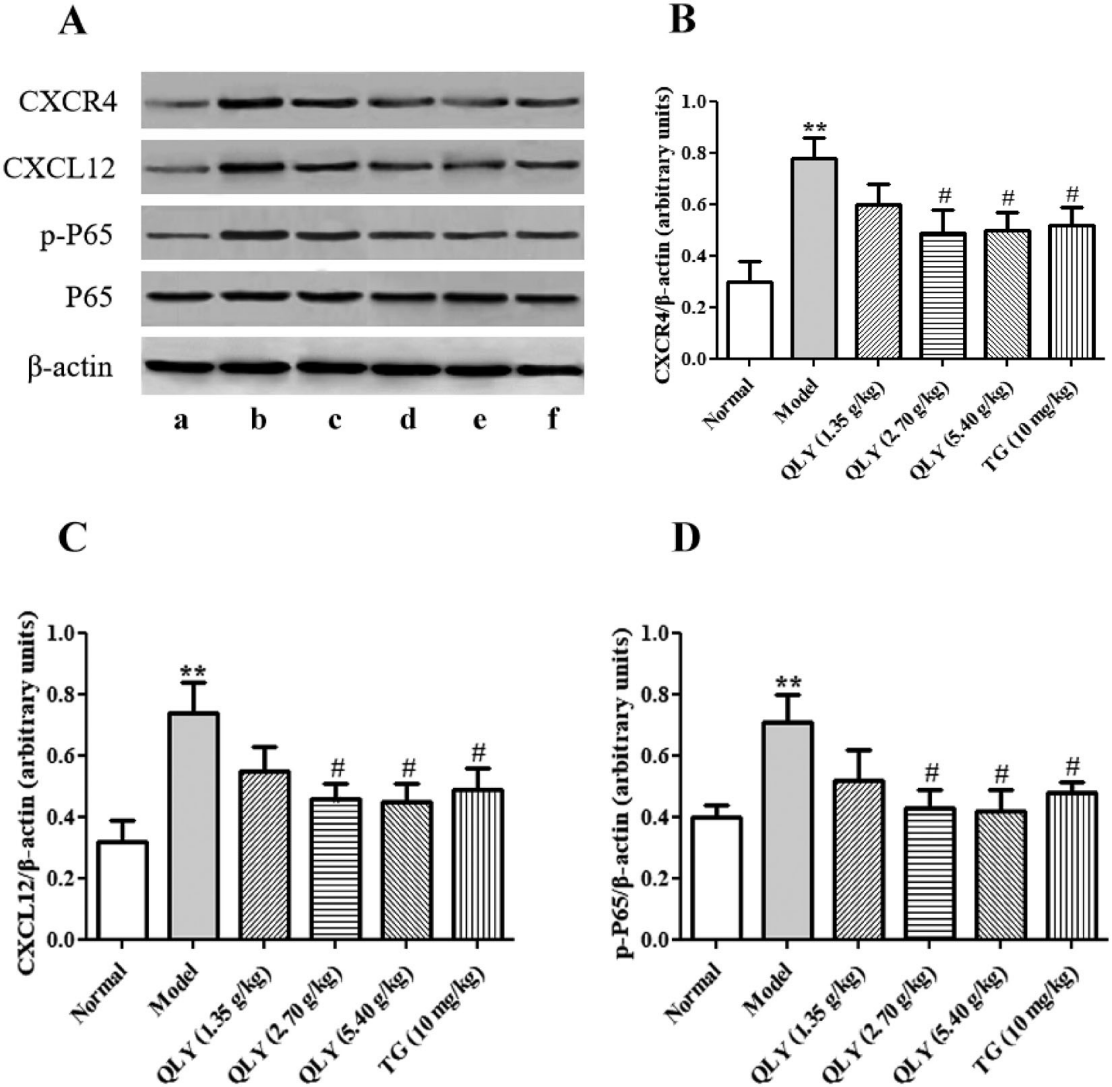
Effects of QLY granules on expression of CXCL12-mediated inflammatory signalling in AA rats. (A) Representative images of Western blotting of the expression of CXCL12, CXCR4, P65 and p-P65. a: normal; b: model; c: QLY granules (1.35 g/kg); d: QLY granules (2.70 g/kg); e: QLY granules (5.40 g/kg); f: TG (10 mg/kg). Western blotting semi-quantification of (B) CXCR4 and (C) CXCL12. (D) Ratio of p-P65 / total P65. Data are expressed as the mean ± SD, with 3 samples in each group. **p < 0.01 vs. normal, ^#^*p* < 0.05, vs. model.

The expression of CXCL12/CXCR4, NF-κB in synovial tissue was detected by Western blotting. As shown in Figure 8, the CFA-immunization not only caused significant CXCL12 and CXCR4 but also increased p-NF-κB p65 as compared to the normal group (*p* < 0.01). The treatment of QLY granules (2.70 and 5.40 g/kg) could markedly inhibit CXCL12, CXCR4 and p-NF-κB p65 in synovium compared with the model group (*p* < 0.05).

**Figure 9.**
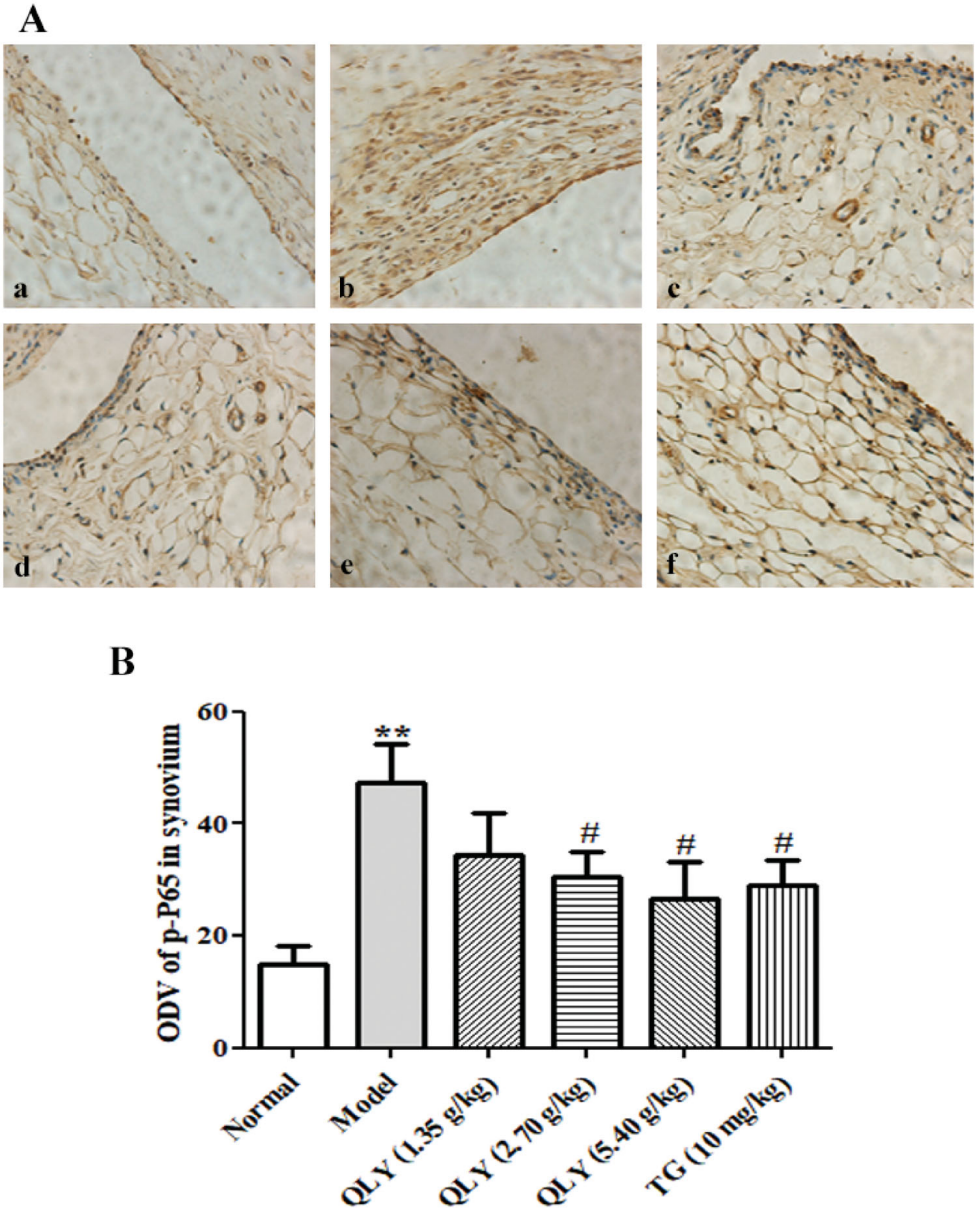
Effects of QLY granules on expression of p-P65 in synovial tissues. (A) Representative immunohistochemical analyses of p-P65 expression in the synovial tissues, illustrating alterations in the synovial tissues of each group of rats (magnification, ×400). a: normal; b: model; c: QLY granules (1.35 g/kg); d: QLY granules (2.70 g/kg); e: QLY granules (5.40 g/kg); f: TG (10 mg/kg). (B) ODVs of p-P65 in the synovial tissues markedly decreased in rats with AA following administration of QLY granules and TG. Data are expressed as the mean ± SD, with 3 samples in each group. **p < 0.01 vs. normal; ^#^*p* < 0.05 vs. model.

To further elucidate the mechanisms of QLY granules exerting effects on AA rats, the expressions of p-NF-κB P65 in the synovium of rats were analyzed by immunohistochemistry. As shown in Figure 9, the results were consistent with those of Western blotting.

## Discussion

The current study examined the anti-arthritic activities of QLY granules, a traditional Chinese medicine (TCM) formula used in the treatment of Hot Syndrome-related rheumatoid arthritis (RA). Network pharmacology studies (Zhang et al. 2013) have found that QLY exerts anti-rheumatoid arthritis effects through inhibiting angiogenesis and inflammatory response. In addition, the TCM compatibility emphasizes Sovereign-Minister-Assistant-Envoy (*Jun-Chen-Zuo-Shi* in Chinese) with proper herbs for enhancing efficiency and reducing toxicity (Fan et al. 2006). The *Jun* herb, *Sophora flavescens,* targets the main causes of RA, for example, inflammatory response, immune response, and angiogenesis. The *Chen* herb, *Sinomenium acutum*, serves to augment the anti-inflammatory and antiangiogenesis effects of *Jun*. *Phellodendron chinensis* has anti-inflammatory, anti-gout effects, which relieves the symptoms of RA during active periods. *Phellodendron chinensis* and *Dioscorea collettii* are *ZuoShi* herbs, which can be used to modulate the therapeutic effects of *Jun-Chen* herbs, counteracting the side effects of *Sophora flavescens* possibly by targeting some off-target genes. It has been detected that (Yang 2010) the main bioactive ingredients in QLY could be matrine, berberine, sinomenine, and their derivatives, as they are main chemical ingredients from major components in QLY, which have direct access to the circulation after oral administration.

RA is a chronic inflammatory disease that affects the small joints of the hands and feet, eventually leading to disability and decreased quality of life (Smolen et al. 2016). Rheumatoid synovial inflammation involves immune cell infiltration, hyperplasia and neoangiogenesis (del Rey et al. 2009; Choudhary et al. 2018). The rat model of AA has found its wide application in preclinical studies to simulate the clinical, pathological, and histological features of RA (Chen et al. 2014). The arthritic aetiology of AA and RA exhibits common pathological and immunological features, including the involvement of inflammatory mediators, immune dysfunction, and pannus formation (Gou et al. 2018). In the present study, the treatment of QLY granules significantly attenuated the clinical features of AA, synovial proliferation, and inflammatory scores *in vivo*. These results confirming that QLY shows therapeutic and protective effects in the AA rat model. Thymus and spleen are two important immune organs, which can systematically reflect the immune functional status. It has been reported (Yang et al. 2016) that the thymus and spleen may develop marked enlargement or hyperplasia during RA. In the study, compared with the AA model group, QLY granules significantly reduced the thymus and spleen indexes in AA rats. These results suggest that QLY granules might help to restore the function of immune organs in AA rats. Cytokine-mediated pathways are known to be central to the development of RA (Saleem et al. 2020). IL-6, TNF-α, IL-1β, IL-2 and IFN-γ are proinflammatory cytokines involved in RA, which have been shown to play key roles in the pathological mechanism in RA. Particularly, IL-1β was found to be the main mediator of cartilage and bone destruction (Joosten et al. 1999). Hence, QLY’s inhibition of IL-1β, IL-6, TNF-α, IL-2 and IFN-γ levels on AA rat serum might be part of the underlying mechanisms for QLY’s anti-rheumatoid arthritis effects.

CXCL12/CXCR4 plays a pivotal role in the development of RA. The CXCR4 and CXCL12 expression levels in serum and joint synovial fluid of the study group were found to be markedly higher than those of controls (Peng et al. 2020). The level of CXCL12 decreased in the serum of patients with RA after synovectomy (Kanbe et al. 2004). CXCR4 is a 7-transmembrane G protein-coupled receptor (GPCR) that only has CXCL12 as a chemokine ligand. Once CXCL12 binds to CXCR4, the rapid internalization of the activated CXCL12/CXCR4 complex will commence. Subsequently, CXCL12/CXCR4 activates downstream signalling pathways, including AKT (Yin et al. 2019), mammalian target of rapamycin (mTOR) (Ieranò et al. 2014) and nuclear factor (NF)-κB signalling (Lin et al. 2018). NF-κB, a transcription factor involved in the regulation of inflammatory response genes, is implicated in the pathogenesis of RA. In a recent study (Gilston et al. 1997), NF-κB, which mediated the initiation and perpetuation of chronic inflammation, was found to be activated during all the stages of RA. The activated NF-κB induced the transcription of pro-inflammatory cytokines such as TNF-α, IL-1β, and IL-6, culminating in synovitis and cartilage damage (Xiang et al. 2016). Hence, drugs targeting the NF-κB signalling pathway are potential therapeutic strategy against RA, which has shown good safety and efficacy. In the present study, QLY granules treatment could significantly decrease the expression of p-NF-κB p65 in the synovial tissues in affected joints as determined by Western blotting method. In addition, Zhang et al. (2013) reported an inhibitory effect of QLY on NF-κB activity, which further confirmed the results obtained in the present study.

## Conclusions

The data presented here revealed the protective effect of QLY granules against paw edoema in AA rats. In addition, QLY granules reduced the level of pro-inflammatory cytokines, markedly inhibited CXCL12, CXCR4 and p-NF-κB p65 protein expression. In summary, QLY granules have obvious therapeutic effects on AA rats, which may be associated with downregulating CXCL12/CXCR4-NF-κB signalling pathway. QLY granules can be used as a candidate for treating RA. In the next step, we will further study its mechanism of action.
